# Editing *Candida*: Origins and Advances of CRISPR Tools

**DOI:** 10.3390/biom16020245

**Published:** 2026-02-04

**Authors:** Adina Schulze, Katharina Kainz, Maria A. Bauer, Didac Carmona-Gutierrez

**Affiliations:** 1 Institute for Molecular Biosciences, NAWI Graz, University of Graz, 8010 Graz, Austria; adina.schulze@uni-graz.at (A.S.); katharina.kainz@uni-graz.at (K.K.); maria.bauer@uni-graz.at (M.A.B.); 2 BioHealth Graz, 8010 Graz, Austria

**Keywords:** pathogenic fungi, Candida, CRISPR, genetics, gene editing, genetic manipulation

## Abstract

Pathogens causing candidiasis encompass a diverse group of ascomycetous yeasts that have become essential models for studying fungal adaptability, pathogenicity, and host–pathogen interactions. Although many candidiasis-promoting species exist as commensals within host microbiota, several have acquired virulence traits that enable opportunistic infections, positioning them as a leading cause of invasive fungal disease in humans. Deciphering the molecular and genetic determinants that underpin the biology of organisms responsible for candidiasis has long been a central objective in medical and molecular mycology. However, research progress has been constrained by intrinsic biological challenges, including noncanonical codon usage and the absence of a complete sexual cycle in diploid species, which have complicated traditional genetic manipulation. CRISPR-Cas9 genome editing has overcome many of these limitations, providing a precise, efficient, and versatile framework for targeted genomic modification. This system has facilitated functional genomic studies ranging from single-gene deletions to high-throughput mutagenesis, yielding new insights into the mechanisms governing virulence, antifungal resistance, and stress adaptation. Since its initial application in *Candida albicans*, CRISPR-Cas9 technology has been refined and adapted for other clinically and industrially relevant species, including *Nakaseomyces glabratus* (formerly referred to as *Candida glabrata*), *Candida parapsilosis*, and *Candida auris*. The present work provides an overview of the evolution of genetic approaches employed in research directed against candidiasis-associated species, with a particular focus on the development and optimization of CRISPR-based systems. It highlights how recent advancements have improved the genetic tractability of these pathogens and outlines emerging opportunities for both fundamental and applied studies in fungal biology.

## 1. Introduction

Candidiasis-associated species comprise a group of ascomycetous yeasts widely distributed in both environmental and human-associated microbiota, many of which reproduce asexually by budding and may undergo morphological transitions that contribute to virulence [1,2]. While over 400 species have been described, only a subset are prominent human opportunistic pathogens. For instance, candidiasis infections caused by *Candida albicans* and increasingly also by pathogens commonly known as non-albicans *Candida* (NAC) species, pose major global medical challenges [3,4]. Of note, although *Nakaseomyces glabratus* (formerly classified as *Candida glabrata*) is no longer classified within the genus *Candida* due to significant genetic divergence [5,6], it remains a causative agent of candidiasis. These organisms can lead to bloodstream infections (called candidemia) or other forms of invasive candidiasis. On a worldwide scale, invasive candidiasis is estimated to affect around 1.56 million people each year, with nearly one million deaths reported, yielding a crude mortality rate of approximately 64% [7]. For candidemia specifically, large cohort studies have found 90-day all-cause mortality rates around 42% in many settings, with attributable mortality of approx. 28% [8]. Although *C. albicans* remains the most frequently isolated species in many regions, a steady shift toward NACs is evident worldwide [9].

The medical implications of this epidemiological picture are substantial. High mortality is compounded by frequent delays in diagnosis, limited antifungal penetration in certain sites, the presence of biofilms on medical devices, and the rise in antifungal resistance, especially among NACs. Additionally, healthcare-associated burden, substantial cost, and the increasing prevalence of at-risk populations, including the elderly, amplify the global health threat. The convergence of high incidence, high mortality, limited therapeutic options and evolving species distribution stresses the urgent need for improved surveillance, rapid diagnostics, and novel antifungals [10].

Despite substantial progress in sequencing and comparative genomics, our understanding of the genetic architecture underlying pathogenicity, stress tolerance, and antifungal resistance in species responsible for candidiasis remains remarkably limited. The majority of available data derive from *C. albicans*, leaving many NACs only partially characterized at the functional genomic level [11]. Moreover, key biological features of candidiasis-associated yeasts, such as their diploid or aneuploid genomes and parasexual reproduction, pose major obstacles to classical genetic manipulation and mutagenesis approaches [12]. This also includes the noncanonical translation of the CUG codon (translated as serine instead of leucine) in so-called CTG-clade species like *C. albicans*, *C. auris*, *Candida dubliniensis*, *C. lusitaniae*, *Candida parapsilosis* or *Candida tropicalis.* As a result, only a fraction of genes predicted to influence antifungal resistance or host interaction have been experimentally validated, and even basic genotype–phenotype relationships remain elusive for most species [11]. This lack of functional genetic knowledge directly limits our capacity to design new therapeutic strategies or diagnostic tools. To bridge this gap, there is an urgent need to expand the toolkit for precise genetic modification across candidiasis-associated yeast species.

In recent years, the advent of genome editing based on the clustered regularly interspaced short palindromic repeats (CRISPR) technology has opened new possibilities for functional genetics in candidiasis-associated yeast species, offering unprecedented precision and efficiency compared to earlier approaches such as the URA Blaster, URA Flipper, Cre-loxP, and UAU1 cassette systems. Early advances in *C. albicans* genetics led to the development of the URA Blaster, which employed the *URA3* gene flanked by hisG sequences from *Salmonella enterica* Typhimurium to facilitate sequential allele deletions through spontaneous recombination [13,14]. Although powerful for generating homozygous deletions in diploid *C. albicans*, its low recombination frequency and the residual hisG sequences left at target loci could interfere with adjacent gene expression or promote unwanted recombination events [15]. To address these limitations, the URA Flipper system was developed, integrating the site-directed FLP/FRT recombination system under an inducible SAP2 promoter. This system is based on the recombination of sequences between short flippase recognition target (FRT) sites by the recombinase flippase (FLP), thus enabling efficient marker excision and leaving behind only a minimal FRT scar [16]. Similarly, the Cre-loxP system allowed for simultaneous targeting of both alleles using loxP-flanked markers and an inducible Cre recombinase, although it remained limited by multiple transformation steps and the requirement for triple auxotrophic strains [17]. The UAU1 cassette, a modified URA Blaster construct containing an *ARG4* marker within *URA3* repeats, further streamlined homozygous deletions by allowing simultaneous selection of both alleles [18]. In later developments, drug resistance markers such as *SAT1* and Ca*NAT1* replaced auxotrophic markers, enabling genetic manipulation of clinical isolates [19,20,21].

These traditional methods have been instrumental in advancing our understanding of candidiasis-promoting pathogens. They laid essential foundations for dissecting gene function and understanding fundamental aspects of fungal biology and virulence. Importantly, these approaches remain highly relevant today. For instance, large-scale mutant collections, generated with classic genome-engineering strategies, provide powerful tools for functional genomics screens aimed at identifying genes required for key biological processes such as morphogenesis and filamentation and determinants of fungal viability [22,23]. A major functional genomics platform developed for *C. albicans* is the Gene Replacement and Conditional Expression (GRACE) library, which is composed of heterozygous gene disruption strains in which transcription of the remaining intact allele is placed under the control of a doxycycline-inducible repressible promoter system [22]. Initially, the library encompassed engineered strains corresponding to 2326 distinct loci in *C. albicans* and is constantly being expanded [22]. However, the traditional methods used to create those libraries are often labor-intensive, time-consuming, and limited in scope. For instance, they require multiple transformations, rely on auxotrophic backgrounds, and are inefficient for large-scale or multi-gene deletions, which constrains their use, e.g., in NACs and clinical isolates [24]. CRISPR technology has the potential to overcome many of these challenges by enabling rapid and targeted genetic manipulation, thereby accelerating the dissection of cellular processes and the identification of novel therapeutic targets. While CRISPR applications in the *Candida* genus and related species have been reviewed previously [12], the field is evolving rapidly, with novel and complementary methodologies continually emerging. This minireview focuses on the development of current applications of CRISPR in causative agents of candidiasis, emphasizing how this toolkit is reshaping genetic engineering in these complex fungal pathogens.

## 2. CRISPR-Cas Technology

**Figure 1:**
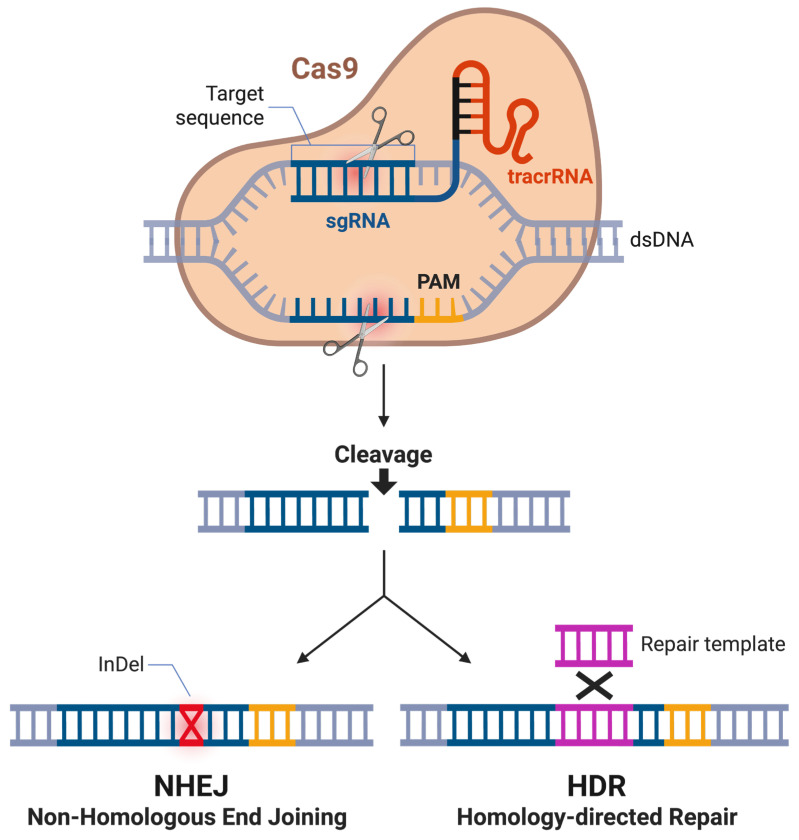
Schematic overview of CRISPR-Cas9-mediated genome editing. Cas9 introduces a site-specific double-strand break in the target DNA. In the absence of an exogenous repair template, the break is predominantly repaired by non-homologous end joining (NHEJ), frequently resulting in insertions or deletions (InDels) that can disrupt gene function. In the presence of a homologous repair template, the break can be repaired through homology-directed repair (HDR), enabling the precise introduction of defined genetic modifications. Figure created in BioRender BETA. Odabas, M. (2026) https://BioRender.com/k6d4x83 (accessed on 26 January 2026).

The CRISPR-Cas (CRISPR-associated) system is a naturally occurring adaptive immune mechanism in bacteria and archaea, where CRISPR-derived RNAs (crRNAs) convey the silencing of invading nucleic acids from viruses and plasmids [25,26]. In brief, the CRISPR locus functions as a molecular memory bank, where short fragments of foreign DNA, known as spacers, are integrated into the host genome between repetitive sequences following infection [27]. Upon subsequent exposure, these spacers are transcribed and processed into crRNAs that guide Cas endonucleases to complementary sequences in invading genetic material, facilitating targeted degradation [27]. This adaptive process allows bacteria to mount sequence-specific immunity against recurrent infections and provides heritable resistance to phage attack [27]. In 2012, the Charpentier and Doudna labs presented a repurposed version of this system to enable targeted genome editing by directing it to specific genetic loci for modification [25] (Figure 1). The engineered system depends on two primary components reminiscent of the natural mechanism: the Cas endonuclease protein and a synthetic guide RNA (sgRNA). The sgRNA binds and steers the Cas enzyme to the desired genetic location, where the enzyme induces a double-stranded break (DSB) in the genome [28]. To fulfill its dual function of guiding the Cas enzyme to the target genomic locus and facilitating enzyme binding, the sgRNA is composed of two key components: the crRNA, which directs specificity through complementary base pairing with the target DNA sequence, and the trans-activating CRISPR RNA (tracrRNA), which is essential for Cas enzyme binding and the formation of an active complex [29]. A successful targeting event also depends on the presence of a protospacer adjacent motif (PAM), a short, conserved DNA sequence adjacent to the target site. The PAM ensures that the CRISPR machinery differentiates between the target and the sgRNA sequence [30].

After the targeted introduction of a DSB, CRISPR-mediated genome editing leverages the cell’s natural genomic repair pathways to introduce changes at the DSB site. The two predominant repair mechanisms are homology-directed repair (HDR) and non-homologous end joining (NHEJ) [31]. HDR uses a homologous DNA template to accurately repair the break and incorporate new sequences [31]. This DNA template can be specifically engineered and introduced externally. In contrast, NHEJ reconnects the broken ends without a template, often resulting in insertions or deletions (indels) at the repair site [32]. In recent years, CRISPR-Cas9 has dramatically advanced genetic engineering, revolutionizing editing of prokaryotic and especially eukaryotic genomes—including those of various *Candida* and related species—where prior tools had substantial limitations [33,34,35].

## 3. CRISPR in *Candida albicans*

### 3.1. Approaches to DNA Editing and Control

**Figure 2:**
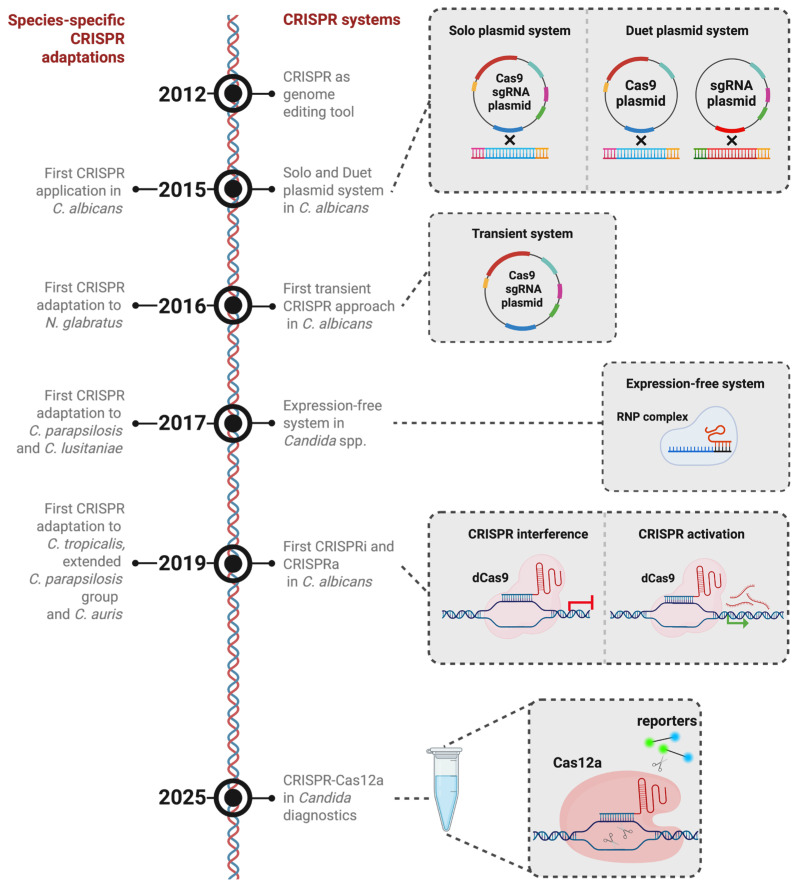
Timeline of CRISPR-based gene-editing innovations in candidiasis-associated species. CRISPR-based gene editing was first reported in **2012**. **(2015)** Initial CRISPR-based strategies in *C. albicans* employed a dual-plasmid configuration, in which the *CAS9* expression cassette and the sgRNA cassette were maintained on separate plasmids and integrated into the genome. This was followed by a single-plasmid system that unified both components into one construct. **(2016)** Subsequently, transient episomal plasmids were introduced as a non-integrative alternative. **(2017)** To minimize species-dependent differences in expression systems, an expression-independent approach using preassembled Cas9-sgRNA ribonucleoprotein (RNP) complexes was later adopted. **(2019)** The implementation of CRISPRi and CRISPRa expanded the toolkit to enable targeted transcriptional repression and activation, facilitating epigenetic modulation. **(2025)** Most recently, CRISPR-Cas12a has been adapted for diagnostic purposes in *C. albicans*. Parallel to these developments, species-specific CRISPR platforms have been engineered to support efficient, cost-effective genome editing across diverse candidiasis-associated yeast species. The components of the CRISPR plasmids are indicated as follows: dark red—Cas9; yellow—Cas9 promoter; cyan—*NAT* resistance marker; green—sgRNA; magenta—SNR53 promoter; blue—*ENO1*; bright red (sgRNA plasmid)—*RP10*. Figure created in BioRender BETA. Odabas, M. (2026) https://BioRender.com/k6d4x83 (accessed on 26 January 2026).

The initial deployment of CRISPR-based genome editing within the *Candida* genus was pioneered in *Candida albicans*, the most thoroughly characterized species. In 2015 [36], the reconfiguration of a CRISPR-Cas was reported for functional use in *C. albicans* (Figure 2, Table 1). Implementing this technology in *C. albicans* necessitated addressing several organism-specific barriers. Notably, some *Candida* spp. or related species translate the CUG codon as serine instead of leucine. As a result, the Cas9 coding sequence required extensive recoding to ensure correct protein synthesis. Moreover, due to the organism’s inability to support autonomously replicating plasmids, the system was built for stable chromosomal integration. For sgRNA transcription, the native RNA polymerase III promoter pSNR52 was employed. Two distinct strategies were established for CRISPR component delivery: a single plasmid co-expressing Cas9 and sgRNA, and a dual-plasmid configuration in which each component was encoded separately. In both approaches, precise gene modifications were achieved by co-delivering an HDR template, typically introducing a frameshift mutation that resulted in premature translational termination. Subsequent genomic analysis revealed that this CRISPR platform enables targeting of more than 98% of *C. albicans* genes, greatly enhancing the capacity for rapid functional genetic studies [36].

However, due to concerns about off-target effects that could result from continuous CRISPR activity, which was designed to remain in the genome, a transient approach was developed to express the system without genome integration (Figure 2) [37,38]. This approach leverages the fact that Cas9/sgRNA-mediated genome editing does not necessitate genomic integration of the expression elements [37]. By restricting Cas9/sgRNA expression to a limited timeframe, this method simplifies the editing process and reduces off-target risks [37]. Moreover, it facilitates simultaneous editing by enabling the delivery of multiple distinct sgRNA constructs into a single cell, allowing for multi-locus targeting [37]. However, despite their considerable efficacy, transient CRISPR-Cas9 approaches are subject to certain constraints. In various model systems, CRISPR-Cas9-based functional genomics screens frequently depend on the formation of small indels through NHEJ to inactivate target genes [39]. Furthermore, transient expression of Cas9 and its associated sgRNAs is currently incompatible with NHEJ-dependent methods, as the expression cassettes are rapidly degraded or become diluted during cell proliferation [37]. Notwithstanding these limitations, the transient CRISPR approach is particularly advantageous for engineering *C. albicans* mutants without leaving selectable markers behind. The scarcity of dominant selectable markers for this species, coupled with the observation that certain markers can influence pathogenicity, makes marker recycling a necessity to study *C. albicans*. For instance, the *URA3* gene, which has been frequently employed as a selection marker in *C. albicans*, critically influences virulence. Consequently, the attenuated virulence observed in strains generated using the ura-blaster cassette cannot be conclusively attributed to the intended gene disruption [14]. Integrating marker excision strategies with CRISPR tools allows for multiple rounds of genome editing in the same background without compromising virulence traits. One such strategy, CRISPR-mediated marker excision (CRIME), induces a DSB within the selectable marker to trigger recombination between flanking direct repeats, leading to the marker’s excision from the genome [38,40]. Further developments, including methods like LEUpOUT and HIS-FLP, have enabled efficient marker-recyclable, homozygous editing in *C. albicans*, while simultaneously allowing for both marker and CRISPR element removal [41]. Using these approaches, entire gene families can be systematically deleted, enabling the characterization of functional differences among individual family members and their collective roles in cellular processes. For instance, deletion of the *TLO* gene family by CRISPR-Cas9 revealed substantial functional diversity, with distinct contributions to carbohydrate metabolism, cell morphology, stress tolerance, and virulence [42].

In 2018, a CRISPR-Cas9-driven gene drive system was introduced, enabling the efficient construction of a comprehensive *C. albicans* double-gene knockout library for systematic genetic interaction studies [43]. Exploiting the organism’s capacity for haploid mating [44], the platform utilizes a so-called gene drive that knocks out a target gene in haploid cells. When these engineered haploids are crossed with wildtype haploids, the gene drive element induces loss of the corresponding wildtype allele in the resulting diploid, thereby producing homozygous deletions. By pairing haploids with each harboring distinct gene deletions, the method facilitates the rapid generation of diploid strains lacking two genes. These double mutants can then be systematically analyzed alongside their respective parental single-gene deletion strains to elucidate genetic interactions and begin mapping the regulatory and functional relationships of the target genes [43,45,46].

Although the aforementioned genome editing strategies are powerful advancements, their applicability is often constrained by the requirement for a CRISPR-Cas9 target site to directly overlap the locus of interest. To overcome this limitation, a novel two-step genome editing approach has been developed [47]. In the first step, Cas9 is guided to introduce a DSB at a defined site close to the genomic feature of interest. This facilitates the replacement of the target site and any intervening sequence by a unique synthetic target sequence termed “AddTag.” In the second step, Cas9 is directed to the AddTag site, enabling the precise insertion of any desired DNA sequence in place of the initially deleted genomic region [47]. This platform, when combined with existing CRISPR engineering methods, enables highly versatile and scarless genome editing without the permanent incorporation of selectable markers [41,47].

### 3.2. Modalities for RNA Targeting and Modulation

Conventional CRISPR platforms typically induce targeted DSBs, followed by repair through a multitude of methods. However, alternative CRISPR systems have emerged that bypass genome cleavage. Among these, CRISPR activation (CRISPRa) and CRISPR interference (CRISPRi) serve as molecular tools to modulate transcriptional activity without altering the target DNA sequence. Earlier advancements in Cas9 engineering revealed that specific amino acid residues can remove its nuclease function while preserving its DNA-binding ability [48,49]. These catalytically inactive variants, referred to as endonuclease-dead Cas9 (dCas9), function as customizable DNA-binding platforms that can recruit effectors to designated genomic sites. When tethered to transcriptional regulators and directed to promoter regions, dCas9 can mediate locus-specific gene regulation [28]. In *C. albicans*, CRISPRi was first demonstrated through two distinct dCas9 fusion strategies [34,50]. One approach utilized dCas9 linked to the mammalian Mxi1 repression domain, resulting in approximately 20-fold gene repression—substantially higher than the ~7-fold repression seen with dCas9 alone [50]. This system proved especially useful in functional studies of essential genes [51]. Notably, downregulation of the essential chaperone gene *HSP90* using this system increased susceptibility to antifungal drugs, in accordance with findings from previous research [50,52]. A second CRISPRi design employed a fusion of dCas9 with the native *C. albicans* transcriptional repressor Nrg1, known for its role in suppressing hyphal development [34]. This construct was guided to the *CAT1* gene encoding catalase, leading to elevated oxidative stress sensitivity and demonstrating effective transcriptional repression in the range of 40–60% [34]. Thus, CRISPRi represents a precise and efficient method for targeted gene repression or functional depletion in *C. albicans* (Figure 2) [34,50].

CRISPRa operates analogously to CRISPRi by utilizing the same catalytically inactive dCas9 carrier. However, instead of recruiting a repressor, CRISPRa directs a transcriptional activator to the promoter region of the target gene. Román et al. [34] achieved this by fusing dCas9 with the transcriptional activation domains Gal4 and/or VP64, targeting the *CAT1* promoter in a manner similar to repressor-based CRISPRi constructs. The resulting CRISPRa strains exhibited increased resistance to hydrogen peroxide and elevated *CAT1* transcript levels, confirming effective transcriptional activation. These findings demonstrate that the dual-activation CRISPRa system is capable of inducing gene expression in *C. albicans*. In 2022, Gervais et al. [53] took advantage of a CRISPRa system in *C. albicans* by exploiting a tripartite activator complex, previously optimized for *S. cerevisiae* [54], fused to dCas9. This system is distinct from other CRISPR-based gene activation platforms developed for *C. albicans* because it utilizes an efficient, single-plasmid design that takes advantage of rapid Golden Gate cloning and thus can streamline *C. albicans* strain engineering. Its broad applicability across various *C. albicans* genetic backgrounds, including clinical isolates, makes it convenient for high-throughput applications. Consequently, this CRISPRa platform could facilitate the generation of large-scale CRISPRa libraries, which already contributed significantly to the understanding of molecular mechanisms in other organisms [55,56,57,58].

### 3.3. Diagnostic Methods

Beyond, a diagnostic method combining CRISPR-Cas12a with thermally driven recombinase amplification has been recently introduced for the detection of *C. albicans* (Figure 2) [59,60]. This approach uses the unique mechanism of Cas12a, which undergoes a structural transformation upon crRNA-directed recognition and binding to a specific DNA sequence. This change initiates its *trans*-cleavage function, enabling degradation of surrounding labeled single-stranded DNA (ssDNA) and thus generating a signal that can be visualized via fluorescence or lateral flow assay platforms. The technique is structured in two independent steps. Initially, target DNA undergoes amplification via enzymatic recombinase amplification (ERA), found to have superior sensitivity [59]. Subsequently, detection is achieved through a CRISPR-Cas12a-mediated *trans*-cleavage reaction. These two processes are integrated into a unified, temperature-regulated, single-tube ERA-CRISPR-Cas12a detection platform [59]. In this configuration, amplification and detection are initially segregated by a wax barrier. The ERA reaction is conducted at 37 °C. Upon completion, the system is heated to 45 °C, causing the wax to melt and enabling the CRISPR-Cas12a detection reagents to merge with the amplification products [59]. This technique proves the versatile applicability of the CRISPR system in *C. albicans* beyond foundational research.

**Table 1:** Overview of published CRISPR-based gene manipulation methods in *Candida albicans*.

Species	Delivery	Activity	Other Features	Reference
*Candida albicans*	Single-plasmid system (Cas9 + sgRNA combined) Dual-plasmid system (Cas9 + sgRNA on separate plasmids)	Genomic integration of CRISPR components at ENO1 locus	Marker Recycling	Vyas et al., 2015 [36]
*Candida albicans*	PCR-derived cassette transformation	Transient expression	Built on Vyas et al., 2015 [36]	Min et al., 2016 [37]
*Candida albicans*	Dual-plasmid system (Cas9 + sgRNA on separate plasmids)	Genomic integration of CRISPR components at ENO1 locus	Marker Recycling, adapted from Vyas et al., 2015 [36]	Ng and Dean 2017 [61]
*Candida albicans*	PCR-derived cassette transformation	Transient expression	Marker Recycling, built on Min et al., 2016 [37]	Huang and Mitchell, 2017 [40]
*Candida albicans*	PCR-derived cassette transformation	Transient genomic integration of CRISPR components at HIS1 locus or LEU2 locus	Marker Recycling	Nguyen et al., 2017 [41]
*Candida albicans*	PCR-derived cassette transformation	Genomic integration of CRISPR components at NEUT5L locus	Gene drive array (GDA)—platform	Shapiro et al., 2018 [33]
*Candida albicans*	Single-plasmid system (Cas9 + sgRNA combined)	Transient genomic integration of CRISPR components at NEUT5L locus	Marker Recycling	Vyas et al., 2018 [62]
*Candida albicans*	PCR-derived cassette transformation	Genomic integration of CRISPR components at ADH1 locus (CAS9) and RP10 locus (sgRNA)	CRISPR interference (CRISPRi, dCas9 repression) CRISPR type: CRISPR activation (CRISPRa, dCas9 activator)	Román et al., 2019 [34]
*Candida albicans*	Single-plasmid system (Cas9 + sgRNA combined)	Genomic integration of CRISPR components at NEUT5L locus	CRISPR interference (CRISPRi, dCas9 repression)	Wensing et al., 2019 [50]
*Candida albicans*	PCR-derived cassette transformation	Transient genomic integration of CRISPR components at HIS1 locus or LEU2 locus	AddTag two-step approach, based on Nguyen et al., 2017 [41]	Seher et al., 2021 [47]
*Candida albicans*	Single-plasmid system (Cas9 + sgRNA combined)	Genomic integration of CRISPR components at NEUT5L locus	CRISPR activation (CRISPRa, dCas9 activator)	Gervais et al., 2023 [53]
*Candida albicans*		Cas12a-based	Diagnostic purposes	Zeng et al., 2025 [59]
*Candida albicans*		Cas12a-based	Diagnostic purposes	Liu et al., 2025 [60]

## 4. CRISPR in NAC Species

Following their initial development in *C. albicans*, CRISPR-based genome editing approaches were gradually adapted to other candidiasis-promoting pathogens, commonly referred to as NAC species. In some cases, CRISPR-Cas9 systems originally optimized for *S. cerevisiae* or *C. albicans* could be directly applied to NAC species without major modification. However, substantial interspecies differences necessitated species-specific adaptations [12].

To overcome the requirement for species-specific expression of Cas9 and sgRNAs via plasmid-based systems, an alternative ribonucleoprotein (RNP)-mediated strategy was developed (Figure 2) [63]. In this approach, recombinant Cas9 protein is assembled in vitro with CRISPR RNAs (crRNA and tracrRNA), and the resulting RNP complexes are delivered into the cells together with donor DNA templates to facilitate homology-directed repair [35,63]. This method demonstrated broad applicability across NAC species and improved transformation efficiencies in several clinical isolates [63]. Importantly, the RNP approach circumvents the need for selectable markers, plasmids, or species-specific promoters, making it particularly advantageous for species with limited molecular genetic tools. Although this system was specifically developed for NAC species, its benefits also render it a good choice for *C. albicans* [64]. Nevertheless, its reliance on purified Cas9 protein causes the approach to be more costly than plasmid-based systems.

As a result, CRISPR-based research in *Candida* spp. and related species has progressed along two parallel trajectories: The development of expression-free, RNP-based platforms and the establishment of species-optimized, plasmid-driven CRISPR-Cas9 approaches [35,63]. In the following, we will discuss the emergence and implementation of these species-specific CRISPR systems (Table 2).

### 4.1. Nakaseomyces Glabratus (Formerly Classified as Candida glabrata)

Unlike *C. albicans*, which actively invades the host, *N. glabratus* appears to rely on a “stealth” strategy within the host, i.e., its ability to persist by evading immunity without provoking strong inflammation [79,80]. Hallmark virulence traits of *C. albicans*, like hyphal differentiation or the secretion of proteolytic enzymes, are absent in *N. glabratus*. Comparative genomics has revealed that *N. glabratus* is evolutionarily more closely aligned with *S. cerevisiae* than with members of the *Candida* CTG clade [81], supporting the hypothesis that human pathogenicity in *N. glabratus* arose via a phylogenetic path distinct from that of other *Candida* spp. [82,83]. Thus, it was possible to rapidly establish CRISPR-based genome editing systems for *N. glabratus* by adapting CRISPR methods originally introduced for *S. cerevisiae* [65]. For instance, unlike in many *Candida* spp, in *N. glabratus*, episomal plasmids remain stable, enabling independent expression of both Cas9 and sgRNAs. To accommodate species-specific transcriptional environments, two alternative sgRNA expression systems have been evaluated. One utilizes the RNA polymerase III-driven SNR52 promoter from *S. cerevisiae*, and the other employs *N. glabratus*’s RNAH1 promoter paired with a tRNA-Tyr-derived terminator. For the Cas9 nuclease, the conventional *S. cerevisiae* TEF1 promoter was replaced with the endogenous pCYC1 promoter from *N. glabratus*. These adaptations to *N. glabratus* significantly improved its viability and increased the efficiency of homologous recombination [65]. The latter is particularly noteworthy, as *N. glabratus* differs from *C. albicans* or *S. cerevisiae*, which predominantly rely on HDR for DNA double-strand break repair; instead, *N. glabratus* employs both HDR and NHEJ [62]. To guide the cell’s repair toward HDR, two different disruption cassettes were successfully transformed as donor templates in parallel with Cas9 and sgRNA plasmids, disrupting the *ADE2* locus and thus paving the way for precise gene modification in *N. glabratus* via HDR [65].

In an effort to streamline the CRISPR-Cas9 technology in *N. glabratus*, Vyas et al. [62] engineered a “unified” plasmid system in which the Cas9 nuclease, sgRNA, and donor DNA are all encoded within a single construct. The so-called “Unified Solo CRISPR system” eliminates the need for separate expression vectors and enables high-throughput mutagenesis and efficient plasmid recycling for repeated rounds of genome editing. Notably, using the “Unified Solo CRISPR” as a framework, an inducible Cas9 construct driven by the *MET3* promoter was introduced, combined with *URA3* as a selectable marker [66,84]. This is interesting, since up to that point, all available CRISPR-Cas9 platforms in *N. glabratus* had relied on constitutive promoters, resulting in continuous expression of Cas9 and sgRNAs immediately after transformation [65]. In contrast, an inducible system separates the transformation from the DNA cleavage, thereby enabling controlled assessment of mutant formation [66]. Accordingly, the mentioned approach of inducible Cas9 expression was able to successfully decouple transformation from Cas9 activation. However, the original plasmid showed instability in *E. coli* due to f1 ori DNA sequence repeats, leading to frequent rearrangements [67]. To overcome this limitation, a redesigned plasmid with improved structural stability was generated. When tested on the *ADE2* locus, the optimized construct yielded nearly complete disruption efficiency via NHEJ, thus inactivating the *ADE2* gene [67].

Besides the strategies based on gene disruption, transcriptional upregulation by employing a catalytically inactive dCas9 fused to transcriptional activators (CRISPRa, see above) has also been investigated in *N. glabratus*. A recent study used a centromeric plasmid carrying dCas9 coupled to the VP64-p65-Rta (VPR) tripartite activation complex [68]. With this system, target genes exhibited enhanced expression ranging from approximately 1.5- to 8-fold above baseline. Moreover, the magnitude of gene induction was shown to depend on the position of the sgRNA within the promoter, providing a titratable transcriptional control [68]. This CRISPR-based tool not only enables in-depth functional genetic analysis in *N. glabratus* but also lays a foundation for efficient generation of large-scale, high-throughput genetic libraries.

### 4.2. Candida parapsilosis

As one of the most relevant species of life-threatening candidaemia due to the rise in azole-resistant variants, *Candida parapsilosis* has been the target of continuous efforts to explore its biology and understand its virulence [85]. In 2017, a transient plasmid-based CRISPR-Cas9 system for gene editing in *C. parapsilosis* was developed, which is applicable even in clinical isolates [70]. The approach uses a codon-optimized *CAS9* under control of the *TEF1* promoter, carried on an autonomously replicating plasmid. The sgRNAs are expressed on the same plasmid via one of two distinct promoters: either from a putative SNR52 RNA polymerase III promoter (*pSNR*) plus a *SUP4* terminator, or from a strong RNA polymerase II promoter (*pGAPDH*) flanked by a 5′ hammerhead (HH) and a 3′ hepatitis delta virus (HDV) ribozyme (*pRIBO*) to generate mature sgRNA. Editing was tested initially at the *ADE2* locus by observing whether transformants displayed the ∆*ade2* phenotype (using the PCR repair template), efficiencies of 10–50% were observed with the *pSNR* strategy yielded efficiencies of 10–50%, the *pRIBO* design achieved 80–100% efficiency [70]. The system was applied to 20 diverse *C. parapsilosis* isolates, and in nearly all strains, *ADE2* editing succeeded with repair template. In many cases, the *ADE2* disruption was also obtained even without repair template (via NHEJ-induced indels). However, additional genes in different strain backgrounds were edited with variable success, ranging from unsuccessful to 100% success rate [70].

The implementation of a transient, plasmid-based CRISPR-Cas9 system with a dominant drug resistance marker circumvents the requirement for auxotrophic strains, thereby enabling genetic manipulation without altering virulence. The downside of the *pRIBO* system is, however, that it is not suitable for large-scale generation of mutant strains. Thus, in 2019, an update of the protocol was published, merging the insertion of the sgRNA sequence in between the two ribozyme sequences into one step [71]. Additionally, the HH ribozyme sequence was replaced by the species-specific tRNA sequence, streamlining the cloning process thanks to its generic usability with a similar efficiency [71]. Notably, this transient CRISPR-Cas9 system has subsequently been adapted for use in species phylogenetically related to *C. parapsilosis*, including *Candida orthopsilosis* [73] and *Candida metapsilosis* [71].

Notably, in diploid species, careful validation of the underlying cause of any observed phenotypic changes is essential. In *C. parapsilosis*, Cas9-induced double-strand breaks have been shown to promote loss of heterozygosity [86]. This process can promote homozygosity in deleterious heterozygous variants, thereby generating unintended gene modifications and thus phenotypic outcomes. Consequently, mutation complementation and the analysis of multiple independent clones remain a critical step even when employing CRISPR-Cas9-based genome editing.

Recently, a PCR-based CRISPR-Cas9 approach was developed to enable rapid fluorescent labeling of *C. parapsilosis* isolates [72]. Using a strategy originally designed for *C. albicans* as a framework [41], the workflow was adapted to integrate eight distinct fluorescent protein-coding genes into donor DNA constructs. The system efficiently generated homozygous knock-in mutants in clinically relevant prototrophic isolates in a single round of transformation. Importantly, targeted modification of the neutral intergenic *CpNEUT5L* locus did not affect growth in rich media, confirming its suitability as a safe harbor site for fluorescent markers. The editing efficiency exceeded 80%, with 20 out of 24 analyzed clones carrying the desired modification. The system allows for selectable markers to be maintained on plasmids or integrating constructs rather than being incorporated into donor DNA. Such marker-recyclable editing represents a major advantage of current CRISPR-Cas9 systems compared with earlier genome modification strategies, including initial CRISPR-Cas9 applications, where residual exogenous sequences remained at the targeted locus [12]. These leftovers often raised concerns about whether observed phenotypes resulted from the intended mutation or from the presence of exogenous elements [87].

In aggregate, two basic CRISPR-Cas9-based genome editing strategies have been established for *C. parapsilosis,* including a plasmid-based system [70] and an integrating approach [72]. Each of these methods offers benefits and limitations, as discussed above. Consequently, the choice of system should be guided by the specific experimental objectives.

### 4.3. Candida tropicalis

Beyond its medical relevance, especially also related to increased antifungal drug resistance rates, *Candida tropicalis* has received considerable attention for its biotechnological potential, particularly in the production of valuable biomolecules such as ethanol, xylitol, and biosurfactants [88,89]. Consequently, the development of efficient and rapid genetic engineering tools for this species is of multifaceted interest. The groundwork for a functioning transient CRISPR-Cas9 system in *C. tropicals* was already laid by previously identified suitable promoters and terminators [90]. Building upon these resources, Lombardi et al. [71] designed CRISPR-Cas9-based systems not only for *C. parapsilosis*, *C. orthopsilosis*, and *C. metapsilosis*, but also adapted prior constructs for application in *C. tropicalis*. They engineered a plasmid in which *CAS9* was expressed under the *Meyerozyma guilliermondii pTEF1* promoter, *SAT1* under the *Candida dubliniensis pTEF1* promoter, and a tRNA-sgRNA-ribozyme cassette positioned between the *Ashbya gossypii pTEF1* promoter and the *S. cerevisiae CYC1* terminator. A key advantage of this construct is its modularity, as most components can be readily exchanged thanks to strategically placed restriction sites. Using this system, highly efficient genome editing was achieved. Introduction of premature stop codons into the *ADE2* locus via HDR templates yielded efficiencies of 88–100% [71]. Of note, in the absence of a donor DNA, NHEJ proved to be highly effective in *C. tropicalis* [71].

Although RNA polymerase III promoters are commonly applied for sgRNA expression, no experimentally validated RNA polymerase III promoters with defined transcription start sites have been identified in *C. tropicalis*. To overcome this limitation and systematically evaluate regulatory elements for *CAS9* and sgRNA expression, eight RNA polymerase II promoters were screened for activity and compatibility. Among these, P_GAP1_ and P_FBA1_ demonstrated the highest expression strengths and were selected for driving *CAS9* and sgRNA expression, respectively. This was the basis for the establishment of two alternative CRISPR-Cas9 platforms [74], one that employed a transient, plasmid-based delivery strategy and another that utilized a genomic integration approach. Using the transient system, single-gene disruptions were achieved with efficiencies ranging from 57% to 100%, while multiplex gene deletions reached approximately 32%. Similarly to the initial plasmid-based CRISPR-Cas9 system reported for *C. tropicalis*, this transient approach induced negligible NHEJ events when a homologous repair template was supplied. Additionally, the present system does, likewise, not require genomic integration of selection markers [74]. In contrast, application of the integrative system, targeted to the *POX4* locus and combined with a repair template, resulted in an HDR efficiency of 100% [74].

As mentioned, the limited availability of RNA polymerase III promoters in *C. tropicalis* has challenged the development of efficient sgRNA expression platforms. To address this limitation, a tRNA:gRNA-based system was established [75]. An endogenous tRNA^Gly^ gene was identified in *C. tropicalis* and utilized as a functional RNA polymerase III promoter to enable the expression of multiple gRNAs for single-gene disruption. Integration of a transient CRISPR-Cas9 cassette fused to the tRNA:gRNA array targeting *URA3*, together with a donor DNA, resulted in successful genome editing in all tested colonies. Following the robust single-gene editing, the system was expanded to express multiple sgRNAs for Cas9-mediated multiplexed targeting. When simultaneously targeting *GFP3* and *URA3*, approximately 71% of colonies displayed the expected dual modifications [75].

Subsequently, the first CRISPRi system in *C. tropicalis* was developed, employing the constitutive *GAP1* promoter and the *ENO1* terminator [74] to drive the expression of a catalytically inactive *Streptococcus pyogenes* dCas9 [75]. To evaluate transcriptional repression, *GFP3* and *ADE2* were selected as reporter genes. Targeting *GFP3* within the coding sequence consistently reduced fluorescence intensity to 34.3–39.1% of control levels. In contrast, repression of *ADE2* varied substantially, ranging from no detectable silencing to 38% expression levels compared to the control, depending on the gRNA target site within the promoter region [75]. Although the CRISPRi system in *C. tropicalis* requires further refinement to achieve consistent repression efficiencies, it demonstrates strong potential for both biomedical and industrial applications.

### 4.4. Candida lusitaniae

Although infections caused by *Candida lusitaniae* generally demonstrate relatively low mortality rates of approx. 5%, this species is being increasingly recognized as a critical clinical challenge due to its emerging resistance to multiple antifungal classes, including amphotericin B, 5-fluorocytosine, and fluconazole [91]. In 2017, the first and, to date, only *C. lusitaniae*-specific CRISPR-Cas9 system was introduced [69]. This transient system is based on two plasmids: one encoding *CAS9* under the constitutive *TDH3* promoter, and another expressing the sgRNA under the RNA polymerase III promoter *pSNR52*. The use of these species-specific promoters proved necessary for efficient gene targeting and was successfully applied to multiple loci in both haploid and diploid cells [69]. To enable HDR, a repair template containing a codon-optimized SAT1 flipper selection marker [20] was provided. Nonetheless, the overall targeting efficiency remained relatively low in both haploid and diploid cells [69]. As in most *Candida* and related species, DSB repair in *C. lusitaniae* can occur through the two competing pathways, HDR or NHEJ. The limited HDR efficiency was therefore attributed to the preferential use of NHEJ. To test this hypothesis, the genes *KU70* and *LIG4*, both essential components of NHEJ, were deleted in a haploid *C. lusitaniae* background. Deletion of *KU70* increased *ADE2* gene disruption efficiency from 25% to 49%, while combined deletion of *KU70* and *LIG4* further enhanced efficiency to 81% [69]. Despite these improvements, NHEJ-deficient mutants have substantial limitations, as disruption of this pathway can impair virulence traits, as previously demonstrated in *C. albicans* [35,92].

### 4.5. Candida auris

The fungal pathogen *Candida auris* often displays multidrug resistance and a significant mortality rate, becoming an urgent health threat, especially in the frame of nosocomial infections [93]. *C. auris* has proven exceptionally difficult to manipulate genetically, driving the adaptation or development of several CRISPR-Cas9-based systems. Each system follows distinct strategies for delivering and expressing Cas9 and sgRNA and for managing selection markers and genomic scars. Of note, these systems vary considerably in efficiency and suitability across different *C. auris* clades.

The first CRISPR approach specifically adapted for *C. auris* was the *ENO1* stable integration system, originally described by Vyas et al. [36] and later applied in *C. auris* by Kim et al. [76]. This system integrates Cas9 and sgRNA expression cassettes at the *ENO1* locus, theoretically allowing continuous Cas9 expression. In practice, it yielded the highest number of transformants in screening experiments but showed very low PCR-verified editing efficiency, averaging only 5.6% [94].

Besides this stable integration system, two temporary strategies were also designed. The *LEU2*-targeting system, known as LEUpOUT, was developed by Nguyen et al. [41] and optimized for *C. auris* by Ennis et al. [77]. In this approach, the CRISPR-Cas9 cassette integrates at the *LEU2* locus, disrupting it during editing. Later, the CRISPR-Cas9 cassette is, however, removed by homologous recombination, restoring *LEU2*. This enables repeated use of the same marker in consecutive transformation rounds. Ennis et al. reported an across-clade average efficiency of 40% for *CAS5* deletion and 99% for *CAS5* restoration at the native locus. Nevertheless, overall PCR-verified correct transformants remained low, at only 5.8% on average, with performance strongly dependent on clade and locus [94].

In parallel to these strategies, which build upon integration, plasmid-based approaches were also developed. The Episomal Plasmid-Induced Cas9 system (EPIC) uses the autonomously replicating sequence Cp*ARS7* from *C. parapsilosis* and has been readily applied to *C. auris* [78]. This system delivers Cas9 and sgRNA from an episomal plasmid maintained under nourseothricin selection, which can be lost upon removal of selection pressure, providing a scarless editing strategy. Genomic epidemiology has identified four independent emergences of *C. auris*, represented by four genetically distinct clades that exhibit varying levels of antifungal resistance and are expected to continue diverging phenotypically [95]. These underlying genetic and phenotypic differences among clades may contribute to the variable editing outcomes observed with the EPIC system. Among all tested systems, EPIC displayed the highest editing accuracy, with an average of 41.9% correct transformants, and efficiencies exceeding 50% in Clade I and III strains. However, the system did not yield any correct transformants in Clade IV backgrounds [94].

The current approaches demonstrate that CRISPR-mediated genome editing in *C. auris* is feasible but remains technically challenging, with system- and clade-dependent differences in efficiency.

## 5. CRISPR-Cas9 as an Accelerator of Candidiasis Research

### 5.1. Pathogenicity of Candidiasis-Associated Species

Since its introduction into the fungal genetic toolbox, CRISPR-Cas9 technology has markedly enhanced the study of candidiasis-associated yeast species by enabling precise and efficient genome editing across multiple isolates. In fact, the CRISPR system has been adapted for diverse purposes beyond gene deletion, including promoter replacement, point mutation introduction, and gene family targeting by using a single sgRNA [36,62]. The flexibility and efficiency of CRISPR-Cas9 have allowed for the systematic dissection of both conserved and species-specific biological processes in candidiasis-related pathogens. This has been (and is) determinant in advancing the understanding of cellular mechanisms that underpin virulence, host interaction, and antifungal resistance.

One of the earliest milestones facilitated using CRISPR in *C. albicans* was the elucidation of genetic networks controlling morphogenesis and biofilm formation. Through large-scale CRISPR-based mutant screens, numerous genes and pathways involved in filamentation were identified, revealing the intricate regulation of this morphological switch [36,96,97,98,99,100,101,102]. Other early CRISPR-related efforts contributed, for example, to conceptualizing that cell cycle arrest may promote hyphal growth and biofilm development independently of major biofilm regulators [103]. These findings represented an important advance, linking fundamental cell biology with virulence-associated morphogenesis.

CRISPR applications rapidly expanded beyond *C. albicans* to other pathogenic species, including *N. glabratus*, *C. parapsilosis*, *C. orthopsilosis*, and *C. auris*, where targeted gene deletions clarified the roles of adhesins in host colonization and virulence [65,73,104,105]. A particularly innovative use of the technology was the simultaneous knockout of entire adhesin gene families by using a single sgRNA that targets conserved regions. This strategy enabled functional dissection of previously redundant adhesin systems [106]. CRISPR-based gene drive systems further accelerated the generation of combinatorial mutants, allowing for the systematic mapping of genetic interactions among adhesins. Shapiro et al. [43], for example, employed such a platform to create a comprehensive library of single and double adhesin mutants in *C. albicans*, identifying epistatic relationships that determined biofilm formation under diverse environmental conditions. These studies demonstrated that virulence traits are often regulated by complex genetic interactions, rather than single genes, highlighting the power of CRISPR-driven genomics to unravel multifactorial phenotypes.

Building on these developments, CRISPR was harnessed to generate combinatorial mutants for metabolic and virulence studies. This is illustrated by studies like the one by Wijnants et al. [107], who used a marker-recycling system to construct double, triple, and quadruple mutants of *C. albicans* sugar kinase genes (*HXK1, HXK2, GLK1, GLK4*), revealing how glycolytic regulation influences adhesion, filamentation, and pathogenicity. This work linked metabolic plasticity to virulence, showing that defects in glycolysis can alter biofilm formation and infection outcomes in murine models. Such comprehensive mutant libraries, made feasible through CRISPR, have fundamentally reshaped the capacity to interrogate pathway interconnectivity in candidiasis-associated yeast biology.

### 5.2. Host–Pathogen Interactions

Beyond pathogenicity, CRISPR systems have also advanced understanding of host–pathogen interactions by enabling precise genetic manipulation of both fungal and host cells. For example, mutants deficient in key regulators of β-glucan masking revealed how *C. albicans* modulates its cell wall composition to evade immune recognition [108,109]. Parallel CRISPR studies in mammalian cells elucidated host defense mechanisms, identifying roles for sphingolipid biosynthesis, C-type lectin receptor signaling, and neutrophil/IL-17F activation in antifungal immunity [110,111,112,113]. Together, these investigations have established CRISPR as a tool to break down the complex interplay between fungal virulence and host immune pathways.

The impact of CRISPR also extends to the field of antifungal drug resistance. By enabling precise genetic validation of resistance-conferring mutations, CRISPR has helped clarify the molecular basis of susceptibility to azoles, polyenes, echinocandins, and emerging antifungal compounds. For instance, targeted editing in *C. lusitaniae* confirmed that a single amino acid substitution in the transcription factor *MRR1* conferred resistance to both fluconazole and 5-fluorocytosine through activation of the efflux transporter *MFS7* [114]. CRISPR-based editing in *N. glabratus* also verified that valine-to-alanine substitutions in Gwt1, an important enzyme for glycosylphosphatidylinositol anchor biosynthesis and thus structural integrity of the cell wall, decreased susceptibility to the investigational antifungal Manogepix [115]. Furthermore, CRISPR-Cas9 editing was employed to introduce a single point mutation at a conserved phosphorylation site within the cell wall integrity regulator *CAS5*, demonstrating that this alteration disrupts nuclear localization upon caspofungin exposure in *C. auris* [77]. More recently, CRISPR-based functional analyses allowed for the investigation of mechanisms underlying *C. auris* Amphotericin B resistance and its associated fitness costs. Using targeted mutagenesis, researchers confirmed that modulation of the sterol biosynthesis genes *ERG3, ERG6, ERG10, ERG11, HMG1*, or *NCP1* confers Amphotericin B resistance, thereby providing direct genetic validation of the mutations driving this clinically significant phenotype [78].

Beyond validating known mechanisms, CRISPR has been instrumental in identifying novel antifungal targets. As an example, CRISPR-mediated deletion of *CDC8* and *CDC43* homologs, corresponding to *S. cerevisiae* genes essential for DNA replication and morphogenesis, respectively, produced severe fitness defects in *C. albicans* and *N. glabratus*, highlighting their potential as antifungal targets [116,117]. The ability to construct conditional alleles and employ CRISPRi systems has further expanded the scope of genetic manipulation to essential genes that cannot be deleted outright. For instance, repression of the chaperone *HSP90* via CRISPRi recapitulated loss-of-function phenotypes, illustrating a strategy to study essential gene function while avoiding lethality [50]. Additionally, the exploitation of CRISPRa systems has expanded experimental possibilities by enabling precise gene overexpression. This approach facilitated detailed characterization of the mitogen-activated protein kinases *STE11* and *SLT2* in *N. glabratus*, revealing that their upregulation enhances tolerance to caspofungin [68]. These methodological strategies have positioned CRISPR not only as a discovery tool but also as a platform for preclinical drug target validation.

Altogether, these examples showcase that CRISPR has significantly advanced the field of genetics in candidiasis-associated species, from the rapid generation of single and combinatorial gene deletions to the functional analysis of essential genes and elucidation of drug resistance mechanisms.

## 6. Conclusions and Future Directions

CRISPR-Cas, which naturally occurs as a prokaryotic adaptive immune system that protects bacteria from bacteriophage infection, has been repurposed as a powerful genome-engineering platform across species. Since its first adaptation to *C. albicans* in 2015, subsequent refinements and species-specific adaptations have rapidly expanded its utility in different candidiasis-associated yeast species, i.e., enabling marker-recyclable genome editing and transient expression systems. Emerging CRISPR-based technologies have further extended the platform’s applications to epigenetic regulation, such as targeted gene interference or activation, and to diagnostic approaches.

Despite the rapid advancements thanks to various CRISPR systems, they are still subject to a range of limitations. CRISPR-Cas genome editing in candidiasis-associated yeast species, in particular, is constrained by both biological and technical limitations that vary across species and strains. The system depends on the induction of DSBs and their repair via host pathways, yet the balance between HDR and NHEJ differs markedly among candidiasis-associated pathogens. For instance, elevated NHEJ activity, as observed in *C. lusitaniae*, or strain- and locus-dependent variability, as in *C. parapsilosis*, leads to inconsistent editing efficiencies [69,70]. Consequently, species-specific optimization, including tailored promoters for Cas9 and sgRNA expression, is required, although suitable regulatory elements remain unidentified for many species [35,61]. Editing efficiency is also locus-dependent and influenced by sgRNA design, repair template configuration, and chromatin accessibility. Secondary structure compatibility of sgRNAs and local nucleosome occupancy can hinder Cas9 targeting, necessitating empirical testing of multiple sgRNAs per gene [118,119,120]. While computational tools such as EuPaGDT or Benchling facilitate sgRNA design and off-target prediction, their advanced applications are largely restricted to well-studied species like *C. albicans* [41,121,122]. Targeting is further limited by PAM sequence requirements [62]. Furthermore, constitutive Cas9 expression and DSB formation can induce DNA damage and cytotoxicity, with stable genomic integration posing greater risks than transient expression systems [35,37]. Despite these limitations, the capacity of CRISPR-Cas to link genotype to phenotype with precision has already markedly accelerated discovery across diverse research domains in the field, including pathogenesis, host–pathogen interaction, and antifungal pharmacology. In fact, the full range of possibilities is expected to unfold as the existing limitations are progressively overcome.

Future developments in CRISPR technologies may even further expand its applicability against candidiasis. This may include, for instance, the tractability of a broader range of candidiasis-associated species, but also the establishment of genome-wide CRISPRi and CRISPRa libraries to enable systematic functional analysis of epigenetic traits. Moreover, in vivo CRISPR approaches may allow for genetic perturbation during infection and real-time dissection of host–pathogen interactions. Further developments may include engineering of candidiasis-associated yeast strains for therapeutic purposes, such as the development of attenuated live vaccines or exploring guide-directed lethal editing of pathogenic fungi.

In any case, CRISPR-Cas-associated innovations have already assumed a transformative role in *Candida* research, enabling rapid and precise genetic studies across a wide range of applications, including basic biology, pharmacological targeting, and diagnostics.

## Figures and Tables

**Table 2:** Overview of published CRISPR-based gene manipulation methods in NAC species.

Species	Delivery	Activity	Other Features	Reference
*Candida lusitaniae*, *Nakaseomyces glabratus* and *Candida auris*	RNA-protein-complex (RNP) electroporation (Cas9 protein + sgRNA)	Expression-free system		Grahl et al., 2017 [63]
*Nakaseomyces glabratus*	Dual-plasmid system (Cas9 + sgRNA on separate plasmids)	Transient expression		Enkler et al., 2016 [65]
*Nakaseomyces glabratus*	Single-plasmid system (Cas9 + sgRNA combined)	Transient genomic integration of CRISPR components at NEUT5L locus Transient expression	Marker Recycling	Vyas et al., 2018 [62]
*Nakaseomyces glabratus*	Single-plasmid system (Cas9 + sgRNA combined)	Transient expression	Marker Recycling	Maroc and Fairhead 2019 [66]
*Nakaseomyces glabratus (*including *Candida bracarensis* and *Candida nivariensis)*	Single-plasmid system (Cas9 + sgRNA combined)	Transient expression	Marker Recycling, adapted from Maroc and Fairhead, 2019 [66] (more stable plasmid also usable in *Candida bracarensis* and *Candida nivariensis*)	Métivier et al., 2024 [67]
*Nakaseomyces glabratus*	Single-plasmid system (Cas9 + sgRNA combined)	Transient expression	Marker Recycling, CRISPR activation (CRISPRa, dCas9 activator)	Maroc et al., 2024 [68]
*Candida lusitaniae*	Dual-plasmid system (Cas9 + sgRNA on separate plasmids)	Transient expression	Based on Min et al., 2016 [37]	Norton et al., 2017 [69]
*Candida parapsilosis*	Single-plasmid system (Cas9 + sgRNA combined)	Transient expression	Marker Recycling	Lombardi et al., 2017 [70]
*Candida parapsilosis, Candida orthopsilosis* and *Candida metapsilosis*	Single-plasmid system (Cas9 + sgRNA combined)	Transient expression	Marker Recycling, adapted from Lombardi et al., 2017 [70], to related species	Lombardi et al., 2019 [71]
*Candida parapsilosis*	PCR-derived cassette transformation	Transient genomic integration of CRISPR components at NEUT5L locus	Marker Recycling, based on Nguyen et al., 2017 [41]	Nemeth et al., 2025 [72]
*Candida orthopsilosis*	Single-plasmid system (Cas9 + sgRNA combined)	Transient expression	Adapted from Lombardi et al., 2017 [70] (adapted to *C. orthopsilosis*)	Zoppo et al., 2018 [73]
*Candida tropicalis*	Single-plasmid system (Cas9 + sgRNA combined)	Transient expression	Marker Recycling, adapted from Lombardi et al., 2017 [70], to related species	Lombardi et al., 2019 [71]
*Candida tropicalis*	PCR-derived cassette transformation	Transient expression Genomic integration of CRISPR components at POX4 locus	Marker Recycling in transient system	Zhang et al., 2020 [74]
*Candida tropicalis*	PCR-derived cassette transformation	Genomic integration of CRISPR components at URA3 locus	Marker Recycling, CRISPR interference (CRISPRi, dCas9 repression), tRNA:gRNA platform	Li et al., 2022 [75]
*Candida auris*	PCR-derived cassette transformation	Genomic integration of CRISPR components	Adapted from Vyas et al., 2015 [36] (adapted to *C. auris*)	Kim et al., 2019 [76]
*Candida auris*	PCR-derived cassette transformation	Transient genomic integration of CRISPR components at LEU2 locus	Marker Recycling, based on system in Nguyen et al., 2017 [41] (adapted to *C. auris*)	Ennis et al., 2021 [77]
*Candida auris*	Single-plasmid system (Cas9 + sgRNA combined)	Transient expression		Carolus et al., 2024 [78]

## Data Availability

Not applicable.

## Abbreviations

The following abbreviations are used in this manuscript:

NAC	non-albicans Candida
CRISPR	clustered regularly interspaced short palindromic repeats
FRT	flippase recognition target
FLP	recombinase flippase
Cas	CRISPR-associated
crRNAs	CRISPR-derived RNAs
sgRNA	synthetic guide RNA
DSB	double-stranded break
tracrRNA	trans-activating CRISPR RNA
PAM	protospacer adjacent motif
HDR	homology-directed repair
NHEJ	non-homologous end joining
indels	insertions or deletions
CRIME	CRISPR-mediated marker excision
CRISPRa	CRISPR activation
CRISPRi	CRISPR interference
dCas9	endonuclease-dead Cas9
ssDNA	single-stranded DNA
ERA	enzymatic recombinase amplification
EPIC	Episomal Plasmid-Induced Cas9 system
GRACE	Gene Replacement and Conditional Expression
